# Identification and functional analysis of non-coding regulatory small RNA FenSr3 in *Bacillus amyloliquefaciens* LPB-18

**DOI:** 10.7717/peerj.15236

**Published:** 2023-05-15

**Authors:** Panping Yang, Chengxin Geng, Shaohui Zhu, Zhen Zhou, Muhammad Bilal, Chengyuan Gu, Hai Xu, Linchun Ji, Benchang Xiao, Jingye Wang, Zhoujie Qian, Li Zhao, Yuping Zhao, Hedong Lu

**Affiliations:** 1 School of Life Science and Food Engineering, Huaiyin Institute of Technology, Huaiyin Institute of Technology, Huaian, Jiangsu, China; 2 College of Food Science and Technology, Nanjing Agricultural University, Nanjing, Weigang, China; 3 National Engineering Research Center for Functional Food, Jiangnan College, Wuxi, Jiangsu, China

**Keywords:** Fengycin, Biosynthesis, sRNA, Transcriptome, Post-transcriptional regulation

## Abstract

*Bacillus amyloliquefaciens* is an interesting microbe in the food processing and manufacturing industries. Non-coding small RNAs (sRNAs) have been shown to play a crucial role in the physiology and metabolism of bacteria by post-transcriptionally regulating gene expression. This study investigated the function of novel sRNA FenSr3 by constructing *fenSr3* deficient strain and complementary strains in* B. amyloliquefaciens* LPB-18 , which were named LPN-18N and LPB-18P, respectively. The result showed significant differences in fengycin yield between strain LPB -18N and LPB-18P. The production of fengycin was significantly enhanced in *B. amyloliquefaciens* LPB-18N, compared with that of the strain LPB-18 from 190.908 mg/L to 327.598 mg/L. Moreover, the production of fengycin decreased from 190.464 mg/L to 38.6 mg/L in* B . amyloliquefaciens* LPB-18P. A comparative transcriptome sequencing was carried out to better understand the complex regulatory mechanism. Transcription analysis revealed that 1037 genes were differentially expressed between* B. amyloliquefaciens* LPB-18 and *B. amyloliquefaciens* LPB-18N, including the key regulatory genes in fatty acid, amino acid biosynthesis, and central carbon metabolism, which could provide sufficient quantities of building precursors for fengycin biosynthesis. The biofilm formation and sporulation was also enhanced in the strain LPB-18N, which indicates that FenSr3 could play a vital role in stress resistance and promotes survival in *B. amyloliquefaciens*. Some sRNAs involved in stress response have been identified in the literature, but their regulatory roles in fengycin production remain unclear. The study will contribute a novel perspective to the regulation mechanism of biosynthesis and the optimization of key metabolites of *B. amyloliquefaciens.*

## Introduction

*Bacillus amyloliquefaciens* is a Gram-positive bacterium that can produce a range of metabolites to inhibit the proliferation of pathogenic bacteria. It also shows application potential in food preservation and feed fermentation(Lai, Luo & Zhu, 2020; WoldemariamYohannes et al., 2020). Fengycin is a class of antimicrobial peptides secreted by *B. amyloliquefaciens,* which consist of a *β*-hydroxy fatty acyl chain and a decapeptide (Lu et al., 2021; Lu et al., 2022). The amphipathic structure facilitates emulsification, solubilization, detergency, and sterilization, thus underscoring its importance in industrial applications (Santos, Silveira & Pereira, 2019). In addition, Piewngam et al. (2018) demonstrated that fengycin reduces colonization of *Staphylococcus aureus* in the intestinal tract by interfering with the quorum-sensing system of the pathogens. Fengycin has promising applications in the field of preservation, biopesticides, and pharmaceuticals (Zhao et al., 2017). However, a poor yield remains a bottleneck for wider commercial and practical applications (He et al., 2021). Through transcriptome and proteome analysis, we previously explored the mechanism of the positive effects of fructose on fengycin biosynthesis (Lu et al., 2021; Lu et al., 2022). Sun et al. (2021) demonstrated that the biosynthesis of fengycin in *B. amyloliquefaciens* fmbJ was positively regulated by the genes *codY*, *comA*, and *spo0A*. The production of fengycin was enhanced by lecithin supplementation in the medium and increased the antagonism effect of *B. amyloliquefaciens* PPL against *Cucumber mosaic* virus (CMV) (Kang, Park & Jung, 2021). However, little is known regarding the biological roles of post-transcriptional events leading to short noncoding RNA in fengycin biosynthesis.

Bacterial small RNAs (sRNA), also known as small non-coding RNAs, are a type of small regulatory RNAs with about 40–500 nucleotides in length. The overwhelming majority of sRNA is combined with the target mRNA by pairing imperfect bases, typically involving 8–10 bases (Storz, Vogel & Wassarman, 2011). Almost all of them have the potential to mechanistically regulate multiple mRNAs and have significant effects on getting involved in adapting to environmental changes and regulatory circuits, such as conditional stress, fatty acid metabolism, quorum-sensing (QS), and biofilm formation (Dutta & Srivastava, 2018; Jorgensen, Pettersen & Kallipolitis, 2020; Liu & Chen, 2022). The main mode of sRNA regulation is in combination with the target mRNA to repress protein biosynthesis through masking ribosome binding site (RBS) or recruiting an RNase E and cleaving in the ribosome-free zones of mRNA (Brantl & Muller, 2021; Kavita, de Mets & Gottesman, 2018). Early termination in a Rho-dependent manner or stabilization of the target mRNA structure is a further outcome of sRNA binding with target mRNA (Ponath, Hor & Vogel, 2022). The chaperone protein Hfq, a multimeric and ring-like structure, plays a vital role in the biofunction of sRNA, including promoting the combination of sRNA with target mRNA and protecting sRNA from RNase degradation (Santiago-Frangos & Woodson, 2018). For example, sRNA fadR-farS-fadE-Hfq constitutes a mixed feed-forward loop that regulates the synthesis pathway and *β*-oxidation cycle of fatty acid in *Vibrio cholerae* (Huber et al., 2020). sRNA AmiL (PA3366.1) is a new QS regulatory sRNA in *Pseudomonas aeruginosa* is that plays a role in global QS-mediated virulence regulation, biofilm formation, elastase activity, hemolytic activity and rhamnolipid production (Pu et al., 2022). sRNA StsR is involved in mediating cell division and growth in the *Rhodobacter sphaeroides* (Grutzner et al., 2021). Therefore, sRNA has multiple regulation functions in bacteria, which is significant in identifying novel regulatory sRNA.

In previous work, we identified a novel sRNA named FenSr3, which was considered as a functional regulatory factor in fengycin biosynthesis. In this study, *fenSr3* was characterized by its knockout and overexpression in *B. amyloliquefaciens* LPB-18, LPB-18N, and LPB-18P. Comparative transcriptome sequencing was conducted to better elucidate the complex regulatory mechanism in biological metabolism. The phenotypic changes were evaluated in wild-type and mutant strains of *B. amyloliquefaciens* LPB-18, including biofilm formation, antimicrobial activity, and sporulation. The findings of the present study will add novel dimensions to the regulation mechanism of biosynthesis and the optimization of crucial metabolites of *B. amyloliquefaciens.*

## Materials & Methods

### Microorganisms and cultivation conditions

The experimental strains and plasmids used in this study are shown in Table 1. *B. amyloliquefaciens* LPB-18 (can produce a series of isoforms of fengycin, fengycin A and fengycin B), and *Escherichia coli* DH5 *α* were cultivated in Luria-Bertani (LB) medium at 33 °C. The strain LPB-18 was grown in LB medium (beef extract 5 g/L peptone 10 g/L, yeast extract 5 g/L, NaCl 5 g/L, and glucose 10 g/L) at 33 °Cfor seed culture. The modified Landy medium was used for Fengycin fermentation. The medium components were: fructose 20 g/L, yeast extract 1 g/L, L-glutamic acid 5 g/L, KCl 0.5 g/L, MgSO_4_ 0.5 g/L, KH_2_PO_4_ 1 g/L, L-phenylalanine 2 mg/L, MnSO_4_ 5 mg/L, FeSO_4_ 0.15 mg/L, and CuSO_4_ 0.16 mg/L. The fermentation was carried out in a 500 mL shake flask containing 200 mL medium at 33 °C with 180 rpm for 72 h. Antibiotics (1 mg/L erythromycin or 5 mg/L chloromycetin for the strain LPB-18, 1 mg/L ampicillin for *E. coli*) were added to the medium appropriately.

**Table 1:** All bacterial strains and plasmids used in this work.

Strain or plasmids	Relative properties	Source
*B. amyloliquefaciens* LPB-18	Wild-type strain produce fengycin	Laboratory stock
LPB-18 Δ*fenSr3*	*fenSr3* deletion strain, a derivative of strain LPB-18	Current study
LPB-18 *fenSr3*	*fenSr3* harbouring pHT- *fenSr3*	Current study
*Escherichia coli* DH 5*α*	competence	Laboratory stock
pCBS	pMAD with minor modification. The 3928–6049 bases in pMAD were removed, and Pamy, SamyE and lacZ were added in that location. Ap^r^ Em^r^ (8102 bp)	Laboratory stock
pCBS-Δ*fenSr3*	pCBS with *fenSr3* deletion box. Apr Emr	Current study
pHT43	*Escherichia coli* and *Bacillus subtilis* shuttle expression vector, Pgrac, Ap^r^ Cm^r^	Laboratory stock
pHT43- *fenSr3*	*fenSr3* expression vector	Current study

### Construction of plasmids and mutation strain

The thermo-sensitive knockout vectors pCBS were obtained from the Nanjing Agricultural University (Sun et al., 2021). All primers used for this study are summarised in Table 2. The mutation construction was performed in the same manner as described previously (Sun et al., 2021). Briefly, the upstream and downstream regions of *fenSr3* were amplified with specific primers. The two fragments were linked by Seamless Cloning/In-Fusion Cloning polymerase chain reaction (PCR) with the primers up-*fenSr3*-F and down-*fenSr3*-R. The integration fragment was inserted into the corresponding restriction sites (EcoRI/BamHI) of pCBS to yield *B. amyloliquefaciens* LPB-18N *via* electrical transformation technology, and the knockout strain of *B. amyloliquefaciens* LPB-18N was validated by PCR amplification using primers up- *fenSr3*-F and down-*fenSr3*-R.

**Table 2:** The primers used in this experiment.

L-FenS-F	atcgatgcatgccatggtaccCCAAGTATCTGTCTCAGAC (Kpn I)
L-FenS-R	GTTCTTCAAGCTCATTGCGGGCAGCCAGCGGGCTA
R-FenS-F	GATTTAGCCCGCTGGCTGCCCGCAATGAGCTTGAA
R-FenS-R	gcgtcgggcgatatcagatctTTATATGTAAGTGACCAGG (Bgl II)
Pgrac F:	caccggaattagcttggtaccCAGCTATTGTAACATAATCGGTACGG (Kpn I)
Pgrac R	TGTAGTAAAGCCATtgatccttcctcctttaattg
NcRNA-F	AggaggaaggatcaGGAGCTGTGTACGCGGTTTGAA
NcRNA- R	gacgtcgactctagaagatctCGGACGGAACGATCGAATATGCCGGACG (Bgl II)
16s rRNA-F	ACGGTCGCAAGACTGAAACT
16s rRNA-R	TAAGGTTCTTCGCGTTGCTT
ComA-F[64304913	GCTCCATCCCATTGACCTC
NZ_CP034943.1]	TTGTCTGTTGATTGTCTCAGTCC
ComA-R	GCAGAAACTCCGCTTGTTG
DegU-F[75093166	GCTGAAAGAGATGGATGCTGAT
NZ_CP072120.1]	TGGCAAGTCATGTTTGGTTT
DegU-R	CGAACTGCGTCGTACTCTTG
AbrB-F[75093757	CAGCCGCGATTTTTACAGC
NZ_CP072120.1]	CGTCATTCCTCTTTCTGTCACAT
AbrB-R	CAACGAGGAAATGGAATCAA
PhrC-	GCGAAGCAATCTCAATGGTAT
F[64302239	TGATAGCGGTCGTGTTA
NZ_CP034943.1]	ACGGTATCCAGGAAGTAG
PhrC -R	TTCTTGATAGCGGTCGTG
Spo0A-F[75092143	GAAGGCGTCTGTCCAT
NZ_CP072120.1]	CGCCTTCTACTTCCTGGATA
Spo0A-R	TGGGCATTGTCGTATTCA
FabD-F[75095156	CAAACGGGACAAGGTA
NZ_CP072120.1]	GACTGAATCGCCAAAG
FabD-R	TTGTGTGATATTGGGATG
FenA- F[17137498	GTCGGGCGATATCGAG
NC_022530.1]	GCCATGGTACCCGGGAGC
FenA-R	CGCGTCGGGCGATATC
FenC-F[17137657	TCCGTAACGGAGACTTC
NC_022530.1]	GCCGTTCTATACGAAGTTC
FenC-R	GAATCCGCTGAGCTCCAGCT
FenD-F[17137658	GATTTCATTACCATCCCG
NC_022530.1]	ATGATGCAGGCAAAGA
FenD-R	CGAAATGTACAAAGCG
TasA-F[5461805	GAATCCATGCCGCTCTT
NC_009725.1]	GGATAAAGAAGACACGGAC

The expression vector was constructed as previously described(Zhang et al., 2017). First, the *fenSr3* was amplified from *B. amyloliquefaciens* LPB-18 with the primers *fenSr3*-F/R and the *Pgrac* promoter was amplified from pHT43 using the primers Pgrac-F/R. The fusion fragment of *fenSr3* and Pgrac were linked by Seamless Cloning/In-Fusion Cloning PCR. After the KpnI and BamHI function, it resulted in pHT-*fenSr3*. Then, the *fenSr3* expression plasmid was transferred into *B. amyloliquefaciens* LPB-18 and the recombinant strain *B. amyloliquefaciens* LPB-18P were validated by PCR.

### Phenotypic assays of wild-type and mutant strains

The dry cell weight was performed to evaluate the effect of non-coding small RNA FenSr3 on cell growth. Different types of strains were cultured, and aliquots were sampled into a pre-weighed tube. After centrifuging at 8,000 rpm for 5 min, the supernatant was discarded, and the cell pellet was collected. The cell pellet was washed three times with normal saline and dried for 5 h to a constant weight at 90 °C. The crude extract was filtered through a 0.22 µm pore filter, and 30 µL of aliquot was injected into an Eclipse XDB-C18 column (5 µm, 250 × 4.6 mm; Agilent Technologies, Santa Clara, CA, USA) in the RT-HPLC system (Shimadzu, Japan) for further identification and purification. The mobile phase was deionized water containing 0.1% trifluoroacetic acid, and mobile phase B was acetonitrile containing 0.1% trifluoroacetic acid. The flow rate was maintained to be six mL/min under the following conditions: 0–15 min, 30% A to 45% A, 70% B to 55% B; 15–55 min, 45% A-55% A, 55% B-45% B. Monitoring was conducted at 205 nm UV detector (Wang et al., 2015). The crude extracts were dried for 4 h to a constant weight at 65 °C. The plate diffusion assays were used to detect the *in vitro* antagonistic activity of wild-type and mutant strains against *Rhizopus* and incubated at 28 °C for 48 h in PDA. The biofilm formation assay was carried out in 96 deep well plates incubating in LB medium at 33 °C for 24 h.

### RNA extraction and transcriptome sequencing

*B. amyloliquefaciens* LPB-18 and *B. amyloliquefaciens* LPB-18N were cultured for 12 h before their RNA was extracted. NCBI_GCF_000262385.1 was used as the reference genome. Total RNA from these strains was extracted using the RNeasy Extraction kit (Invitrogen Life Technologies, Gaithersburg, MD, USA) according to its manufacturer’s instructions. RNA quality was assessed using Nanodrop 2000 (Thermo Scientific, Waltham, MA, USA). Before sequencing analysis, the ribosomal RNAs were wiped using the RiboZero rRNA Removal Kit (Epicentre) for a gram-positive bacterium. The extracted total RNA was used as a template to synthesize the cDNA first strand using HiScript First Strand cDNA Synthesis Kit (Vazyme, Nanjing, China). Subsequently, the cDNA was sequenced by Genedenovo Biotechnology Co., Ltd (Guangzhou, China) using the Illumina sequencing platform (http://www.omicshare.com). EdgeR 4 software was used to analyze the quantity of gene expression differences between groups using FDR and log2FC genetic variations to filter, filter condition for FDR <0.05 and — log2FC —>1. The functional classification analysis was conducted by Gene Ontology (GO) enrichment analysis (http://www.omicsmart.com/RNAseq/home.html), Kyoto Encyclopedia of Genes and Genomes (KEGG) pathway analysis (https://www.omicsmart.com/). Data were collected as previously described in Sun (2021). Specifically, RT-qPCR was used to determine the results of RNA-seq, and the genes of 16s rRNA were used as the reference to normalize the expression of examined genes. The specific procedure of RT-qPCR was performed according to Lu et al. (2022). The relative fold change of the target gene expression was evaluated by the calculation of 2^−ΔΔCt^. The threshold cycle (Ct) values were obtained by the real-time PCR System software.

### Analysis of biofilm formation and spore count

The wild strain LPB-18 and mutation strain Δ *fenSr3* were inoculated into sterile MRS for 5 days at 33 °C. After that, the plates were washed with ddH_2_O, dried at 40 °C for 20 min, and strained with five mL of 0.5% (v/v) crystal violet solution for 3 min. The biofilm on the wells was dissolved with one mL of 95% (v/v) ethanol, and the OD 590 value was measured as their biofilm formation ability (Caro-Astorga et al., 2020). All the samples were analyzed in triplicate. The wild strain LPB-18 and mutation strain Δ *fenSr3* were inoculated in LB medium at 33 °C for 12 h. The spore counting was conducted by plate counting method. Briefly, 100 uL nutrient medium was heated at 65 °C for 30 min. After that, the samples were incubated in culture dish with LB for 24 h, and colonies were counted. Each sample was analyzed in triplicate.

### Statistical analysis

One-way analysis of variance (ANOVA) in SPSS software (SPSS, ver. 17.0, IBM, Armonk, NY) was used for statistical analysis. *P* values were given with the results of ANOVA. The test of Duncan was performed to detect the significance below 0.05. All results were conducted with a triplicate of experiment and mean standard deviation (SD).

## Results

### The characteristic of sRNA FenSr3 and its role in biomass and fengycin production in *B. amyloliquefaciens*

FenSr3 was identified in *B. amyloliquefaciens* as a ∼355 nt sRNA that is considered an important regulatory factor in fengycin biosynthesis. The sRNA is transcribed from an intragenic region of *fenC*, which was the biosynthesis gene of fengycin. The genomic context of this sRNA gene is highly conserved in the family of *B. subtilis* (Fig. S1).

**Figure 1:**
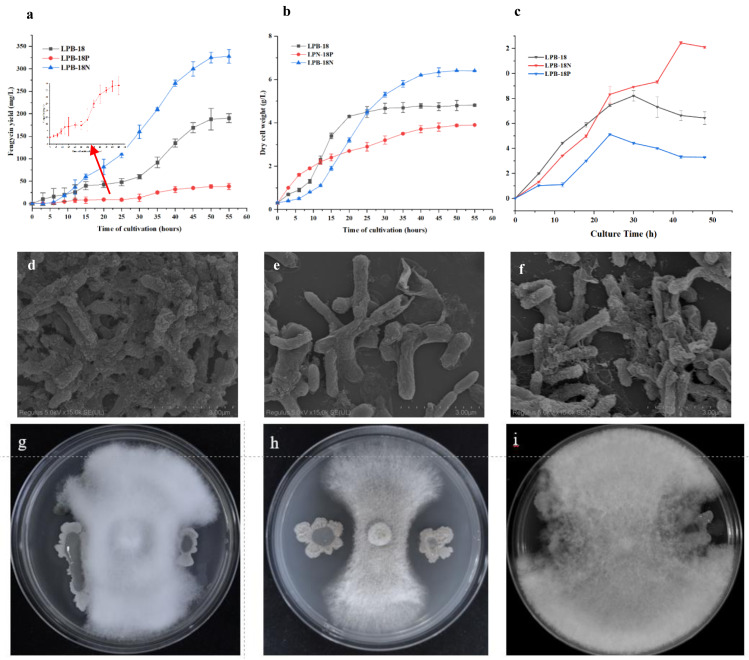
Phenotypic assays and fengycin yield of *B. amyloliquefaciens*. (A) Influence of non-coding small RNA FenSr3 on the yield of fengycin. (B) The growth curve of *B. amyloliquefaciens* in LB media. (C) The biofilm formation of the[i] *B. amyloliquefa*.

The strains were cultivated and regularly sampled to investigate the effect of FenSr3 on the phenotype of *B. amyloliquefaciens*. Fig. 1B displays the result of the determination of dry cell weight. The strain LPB-18 exhibited a standard S-shaped growth curve, which reached a stable phase after about 20 h. After 50 h, the biomass of the strain LPB-18N increased persistently, reaching a maximum biomass value of 6.8 g/L. Additionally, regarding the LPB-18N culture was worth noting that the biomass increased slowly at approximately 12 h and exhibited a more extended logarithmic growth than strain LPB-18 and LPB-18P. As shown in Fig. 1A, fengycin production was significantly increased in *B. amyloliquefaciens* LPB-18N, from 190.464 to 327.5 ± 2.6 mg/L. The strain LPB-18P showed a decreased fengycin concentration of 38.6 ± 9.8 mg/L. In order to compare biofilm development between wild-type and mutant strains, which were consistent with the antagonistic activities of *B. amyloliquefaciens* against *F. oxysporum* in PDA (Figs. 1G–1I). It has already been reported that *B. amyloliquefaciens* could suppress *F. oxysporum* and other filamentous fungi (Wang et al. 2022). The wild-type strain LPB-18 and the mutant exhibited less antagonism against *F. oxysporum* than the *fenSr3*-deficient strain *B. amyloliquefaciens* LPB-18N, as indicated by clear zones in the inoculated bacteria culture. To compare biofilm development between wild-type and mutant strains, the crystal violet assay was performed to evaluate biofilm formation. The wild-type strain and the strain LPB-18P formed moderate biofilms with OD590 nm equivalent to 0.81 and 0.52 in TSB. The strain LPB-18N formed robust biofilms and recorded OD590 nm equivalent to 2.09 (Fig. 1C). Under high magnification, *B. amyloliquefaciens* LPB-18 demonstrated the 3D structure of fold cell surface morphology (Fig. 1D). The micrographs of cell surface also revealed the presence of appendages which were conducive to make the interconnection of biofilm cells and physical surface (Fig. 1E). On the other hand, the strain LPB-18P exhibited less complex and smooth cellular surface (Fig. 1F).

### Transcriptome analysis of *B. amyloliquefaciens* LPB-18N in response to fengycin biosynthesis

**Figure 2:**
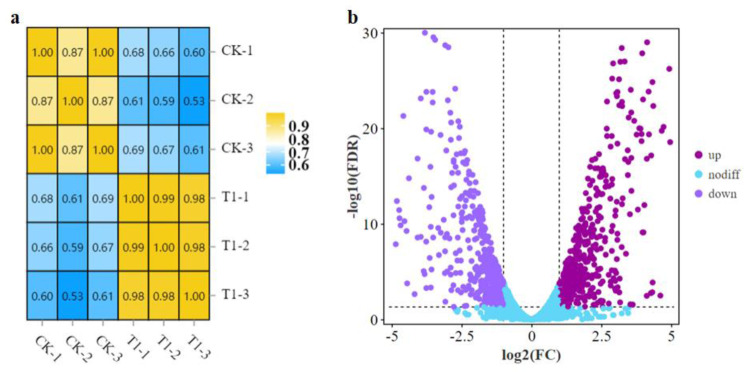
(A) Relationship of expression levels between samples. CK indicates the enrichment of *B. amyloliquefaciens* LPB-18 gene expression. T1 indicates the enrichment of *B. amyloliquefaciens* LPB-18N gene expression level. (B) Transcriptome sequencing.

A comparative transcriptome analysis was performed between strain LPB-18 and strain LPB-18N to comprehensively research the gene expression global changes. As shown in Fig. 2A, the correction between the samples was greater than 0.96 in *B. amyloliquefaciens* LPB-18 and *B. amyloliquefaciens* LPB-18N, which were analyzed by the algorithm of Pearson Correlation Coefficient. This finding demonstrated high repeatability of sample Parallelism and reliability of the transcriptome data. The correction of strain LPB-18 *vs.* strain LPB-18N was 0.65, indicating that sRNA FenSr3 significantly affects the gene expression of *B. amyloliquefaciens* LPB-18. The statistical power of this experimental design, calculated in RNASeqPower, is 0.999882 (https://rodrigo-arcoverde.shinyapps.io/rnaseq_power_calc/). Subsequently, DgeR 4 software was used to summarize difference expression genes (DEGs). According to the criteria of FDR<0.05 and —log_2_FC—>1, 1037 DEGs were screened in *B. amyloliquefaciens* LPB-18N, which included 548 genes were significantly upregulated, and 489 genes were remarkably downregulated. As shown in Fig. 2B, the volcano plots were created to exhibit the different expression genes in wild-type strain LPB-18 and strain LPB-18N, with upregulated genes in red and downregulated genes in green.

**Figure 3:**
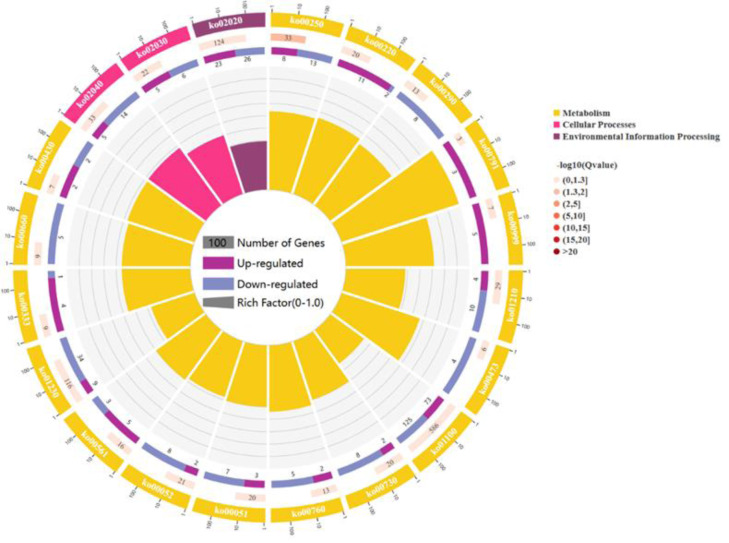
The figure enriched signal pathways that were differentially expressed in the metabolic processes of cellular component and biological process, with yellow representing cellular component and light red representing biological process. A circle graph has four circles from inside to outside. First lap: the classification of enrichment, outside the circle is the scale of the number of genes, and different colors represent different classifications. Second circle: the number of the classification in the background gene and the *Q* or *P* value. The length of the bar indicates the number of genes, and the color depth indicates the size of *P* or *Q* value; the third circle: up-down-regulated gene ratio bar chart, dark purple represents the proportion of up-regulated genes, light purple represents down-regulated gene proportion; Circle 4: RichFactor value for each category (the ratio of the differential genes in the classification to the total number of classified genes).

For a comprehensive describing gene function, *P* < 0.05 was considered the threshold for significant enrichment, and DEGs were enriched in 20 pathways involving 267 genes: 17 pathways for cell metabolic processes, 2 pathway refer to cell processes and 1 pathway participates in environmental signal sensing. (As shown in Fig. 3). In a nutshell, the DEGs enrichment in the biological process includes arginine biosynthesis (ko00220), valine, leucine, and isoleucine biosynthesis (ko00290), flagellar assembly (ko02040), fatty acid biosynthesis (ko02040), biosynthesis of antibiotics (ko01130), secondary metabolites (ko01110) and carbon metabolism (ko01200). The molecular functions enriched for identified DEGs include DNA replication (ko03030), RNA synthesis and degradation (ko03018 and ko03020), and aminoacyl-tRNA biosynthesis (ko00970), and base excision repair (ko03410). The main cellular components enriched for the identified DEGs include cellular protein-containing complexes (ko02040), ribosomes(ko00750), translocator (ko02010 and ko03060), non-ribosomal peptide structures (ko01054), and cell growth and death (ko04112).

### Metabolic pathway analysis of DEGs in the biosynthesis of fengycin

**Figure 4:**
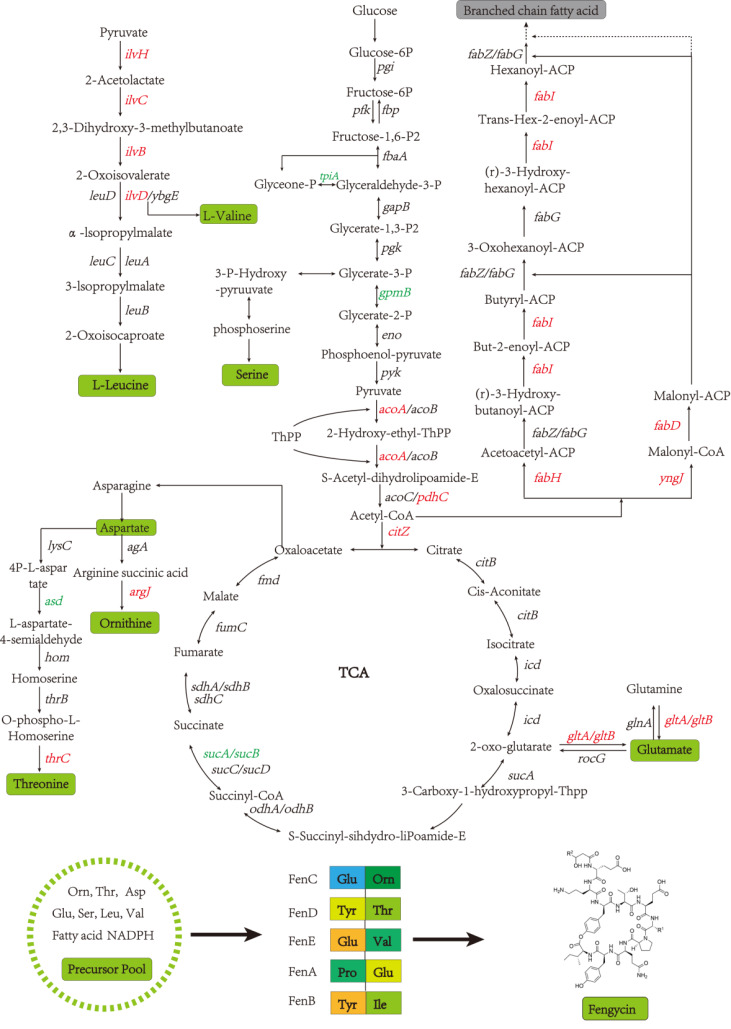
The glycolysis, tricarboxylic acid cycle, amino acid metabolism and fatty acid metabolism of Bacillus amyloliquefaciens LPB-18N were enriched in the graph. Red represents the up-regulation gene and green represents the down-regulation gene compared to the wild type LPB-18.

A global fengycin biosynthesis pathway was constructed in *B. amyloliquefaciens* LPB-18N, as shown in Fig. 4. Changes in individual metabolic processes were analyzed and shown with a metabolic pathway map. A unitive theme of the metabolic processes during fengycin synthesis development was an upregulation of energy-generating and precursor biosynthetic pathways in *B. amyloliquefaciens* LPB-18N. The glycolytic and the tricarboxylic acid (TCA) cycle provide multiple precursors for cellar metabolism and generate a large amount of NADH/NADPH. These positive alterations in metabolite levels are primarily correlated with carbon metabolism and fatty acid synthesis. Specifically, the results revealed the *acoA-acoB-acoC-citZ* operon (encoding pyruvate dehydrogenase system) exhibited an increase in transcript abundance that providing enough precursor in the TCA cycle and branched fatty acid biosynthesis. It is important that pyruvate is a pivotal metabolite linking amino acids and branched fatty acid biosynthesis (Xu et al., 2020). Upregulation of the TCA cycle was consistent with increasing levels of *de novo* nucleotide biosynthesis, including ATP, CTP, and GTP, which could increase the concentration of extracellular DNA (eDNA), a key component of biofilm. The effects of the TCA cycle on promoting biofilm formation had detailed in *B. subtilis* (Pisithkul et al., 2019), and it is consistent with transcript sequencing and biofilm phenotype in *B. amyloliquefaciens* LPB-18N. Besides, a few intracellular branched amino acids also displayed an increasing trend in the transcript, including Orn, Thr, Asp, Glu, Ser, Leu, and Val. The ^13^C-tracer experiments demonstrated the carbon could incorporate into various amino acids and acetylated amino acids at 16 to 24 h in biofilm formation in *B. subtilis* (Pisithkul et al., 2019). The phenotypic experiments and transcript sequencing reveal that carbon cycle may be critical for providing precursors and energy to the cellular process involved in fengycin and biofilm biosynthesis.

Fatty acids are another natural constituent of fengycin, making up the hydrophobic part of fengcyin. A higher expression level of acyl-carrier protein synthase provides enough acyl-carrier protein (ACP), which is dominant in the biosynthesis of fatty acids. Acetyl-CoA is an essential initiator and precursor in the biosynthesis of fatty acids. Interestingly, the genes that participate in the conversion of pyruvate with acetyl-CoA and generation were up-regulated in *B. amyloliquefaciens* LPB-18N, which would be essential for biosynthesizing and assembling fengycin. The deletion of *fenSr3* could affect the synthesis of non-ribosomal peptides, in which fengycin synthesis genes of *fenA*, *fenC,* and *fenD* upregulate 8.91 folds, 7.03 folds, and 8.82 folds, respectively. In addition, the knockout of *fenSr3* could affect carbohydrate metabolism, amino acid, and lipid biosynthesis, which was manifested in better fengycin production and higher expression of genes encoding transport protein, such as ABC transporters (*znuB*, *yhcB*, *opuBA,* and *tcyA*) and lipid transport protein (*estA*, *lcfB*, *plsC*). And the global regulation factor *abrB* (−4.7 folds) was significantly downregulated. According to transcriptome results, we also found that the *comX*, *phrA*, and *phrC* genes were remarkably upregulated, participating in environmental responses in quorum sensing systems. The deletion of non-coding RNA FenSr3 also enhanced the DNA replication and transcription, manifested in responding gene-upregulated including *dnaG*, *dnaE*, *rnhB*, *ygzB,* and *hupA*. After the absence of components that promote the adaptation of bacteria to the challenging environment, bacteria could survive in harsh conditions by enhancing their metabolism and self-repair, which may be a self-defense behavior of bacteria.

### Differentially expressed genes associated with a knockout of *fenSr3* in *B. amyloliquefaciens* LPB-18N

**Figure 5:**
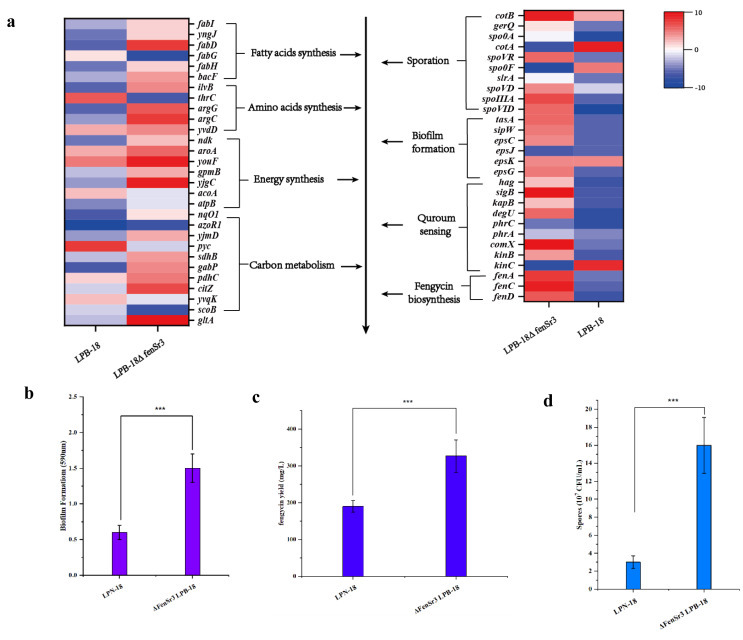
(A) Enriched in eight differential genes of different metabolic processes. Red represents the up-regulation and dark blue represents the down-regulation. 5-b compared fengycin synthesis, biofilm synthesis and spore formation between the strain LPB-18 and ΔfenSr3 LPB-18.

**Figure 6:**
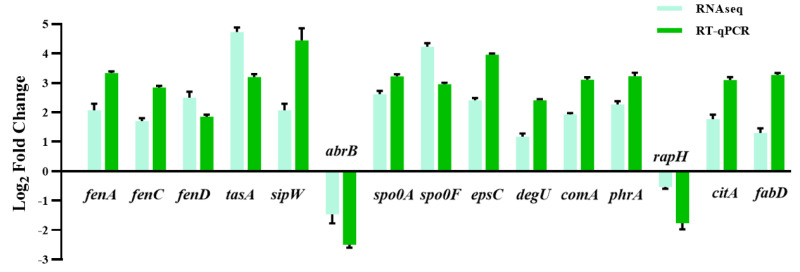
The quantitative fluorescence values of different representative genes were up-regulated in transcriptome sequencing and verification group, and down-regulated in *vice versa*.

The huge distinction in gene expression between strains LPB-18 and LPB-18N was analyzed by metabolic pathway analysis, including spore, biofilm, and fengycin biosynthesis (Fig. 5). According to transcriptome data, the coding gene *abrB* was down-regulation (−2.1 folds) in *B. amyloliquefaciens* LPB-18N. The opposite of fengycin biosynthesis genes *fenA*, *fenC,* and *fenD* exhibited a remarkable increase in deletion strain of *fenSr3*, which encoded fengycin synthetases FenA, FenC, and FenD, respectively. These results were consistent with qPCR, as shown in Fig. 6. Fengycin has an amphipathic structure comprised of a cycle peptide and a hydrophobic fatty acid chain. In addition to the non-ribosomal peptide synthetases (NRPSs), it is also essential to synthesize amino acid and fatty acid residues. In the transcriptome data, nine genes involved in carbon metabolism, including three rate-limiting enzyme genes of glycolysis and tricarboxylic acid cycle (TCA): *pdhD*, *citA,* and *mdh*, which encodes dihydrolipoamide dehydrogenase E3, citroyl synthetase and malic dehydrogenase, respectively, were significantly upregulated. These metabolic processes promote energy supplements and gain.

Also, gene cluster fabG/D/Lmore precursors for the synthesis of amino acid and fatty acid. It was remarkably increased, which encodes ACP S–malonyltransferase and fatty acids synthesis, and there was a great consistency with RT-qPCR (Fig. 6). It indicates good fatty acid synthetic ability in *B. amyloliquefaciens* LPB-18N. The biofilm formation and sporulation were also evaluated (Figs. 5B and 5C). It shows a significant difference between wild-type LPB-18 and strain LPB-18N, which indicates that non-coding small RNA *fenSr3* is essential for biofilm and sporulation.

## Discussion

Small non-coding RNAs are known to play a significant role in gene expression regulation (Ul (Haq, Muller & Brantl, 2020). Recent years have seen a surge in studying the function of sRNA in gene expression, which involves participating in the tolerance of stress conditions and regulation of cellar growth and metabolism (Gerrick et al., 2018). Although many have been identified in *E. coli* and *B. subtilis*, only a few have been deeply investigated. Gene editing technology, such as nucleotide deletions and overexpression, is a popular method of researching gene function. In this study, we verified the biofunction of previously screened small non-coding fragments with the regulation in the biosynthesis of fengycin by structuring knockout and overexpression strain of *fenSr3* in *B. amyloliquefaciens* LPB-18. After detecting fengycin production in different stages, FenSr3 has been shown to have adverse effects on the biosynthesis of fengycin, and the yield was enhanced in *B. amyloliquefaciens* LPB-18N. With prolonged logarithmic and stationary phases, the efficient production of fengycin could indicate high bacterial biomass and high metabolic activity. As a secondary metabolite production, fengycin is mainly secreted in the stationary phase and regulated by multiple signal factors. The significant distinction in mutant strains involves changing with cell division, manifested by the biomass in different phases. The strain LPB-18N exhibited the longest logarithmic phase and had the biggest biomass after 55 h cultivation. Tight regulation of cell division is significant for survival in various environments for bacteria (Pucci et al., 1997). Grutzner et al. (2021) demonstrated the important role of small non-coding sRNA StsR in the cell division of *Rhodobacter sphaeroides.* StsR binds to target sRNA UpsM as well as to the 5 ′UTR of target gene *dcw* (cell wall synthesis) mRNA, causing a conformational change that, in turn triggers cleavage by RNase E, and consequently affecting the level of *dcw* mRNA and cell division. After blast comparison, it was found that *fenSr3* is located inside fengycin syntheses gene *fenC.* Combined fengycin production of wild-type and *fenSr3*-knockout strain, it is reasonable to assume that FenSr3 is involved in either the premature termination of transcription or reducing the stability of mRNA of FenC. There was also a notable difference in the growth pattern of three types of strain in the first 12 h. It could be a mechanism of environmental responsibility, suggesting that FenSr3 may be a responder to different environments. The cell of *B. amyloliquefaciens* LPB-18N could get sensitive to environmental factors, and systemic resistance would be triggered, promoting biofilm formation, sporulation, and secretion of antibiotics to improve resistance to harsh conditions and cell survival rate. After all, the formation of spores and biofilms is essential to better survival of the population in adaptation to changing harsh environments for *B. subtilis* (Qin, Angelini & Chai, 2022). Biofilms are a mode of assemblages of multicellular communities encapsulated by a self-produced extracellular matrix that encloses the surface and confers properties to protect against environmental stressors (Arnaouteli et al., 2021). For Bacillus species, spore formation is a manner to survive upon starvation, and the spore can awaken when the environment becomes comfortable (Xing & Harper Jr, 2020).

**Figure 7:**
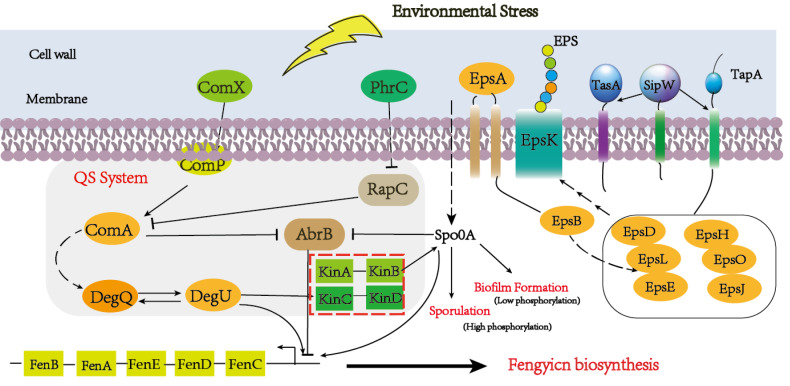
A hypothetical regulatory model for inducing the synthesis of fenSr3 modulators and three macromolecules (fengycin, biofilm, and spore) in Pentyl Bacillus LPB-18.

Quorum-sensing (QS) signaling, a global regulatory mechanism, is crucial for regulating different subpopulations of cell–cell communication in a stress condition (Mulligan, 2005; Pacwa-Plociniczak et al., 2011). The QS system has multiple protein factors, including ComA, ComP, PhrA/C, DegQ/U, and so on. ComX is the precursor of ComQ, when the ComQ prenyltransferase modified ComX, the sensor kinase ComP can recognize it at a certain concentration (Treinen et al., 2021). After triggering Quorum sensing, DegU and PhrA/C were respectively activated and responded to the extracellular signal to promote the metabolism of downstream substances (Stein, 2005). The global transcription regulator AbrB is a DNA-binding protein which could repress multiple genes during the stationary phase and regulate the sequence of events, including cell motility, sporulation, antibiotics production, competence, and cannibalism (Kobir et al., 2014). In our result, comX and phrC were significantly up-regulated, and their regulation pathways are shown in Fig. 7. At the same time, we also analyzed that the encoding gene of DegU was a high expression, which was consistent with previous reports that DegU exerts positive effects on the biosynthesis of fengycin (Sun et al., 2021). In the previous report, DegU/P had been demonstrated to regulate multiple genes, including flagellin proteins and non-ribosomal peptide synthetases. The positive regulation of DegU with plipastatin, a member of the fengycin family, had been proven by point mutants in the DegQ promoter (Lilge et al., 2021). A consistent result was obtained with increased fengycin yield and up-regulated expression synthetases of fengycin in transcriptome analysis. In the knockout strain of *fenSr3*, the up-regulated expression of degU attributes to the government of quorum sensing system or direct regulating by non-coding small RNA would be further investigated. The increased biosynthesis of amino acids and branched fatty acids provide sufficient components with fengycin. As an important composition of membrane lipids, fatty acid also plays an important role in remaining membrane integrity and giving abundance biophysical property of membranes, such as permeability to solutes, membrane fluidity and protein-protein interaction, which are significant to adopt to changing environment by rapid modifying the fatty acids that embed in the membrane (Zhang & Rock, 2008). Previous studies have revealed that cyclopropane fatty acid synthase gene *cfa* was regulated by sRNA RydC, ArrS and CpxQ. The activate expression of *cfa* through different post-transcriptional regulatory mechanisms, sRNA RydC stabilizes *cfa* mRNA and sRNA ArrS and CpxQ affect through overlapping RNase E cleavage site in the *cfa* mRNA 5 ′ UTR (Bianco, Frohlich & Vanderpool, 2019). In *B. amyloliquefaciens* LPB-18, the biosynthesis pathway of fatty acid exhibits significant regulation, which indicates sRNA FenSr3 plays a vital environment responding.

## Conclusions

This study provides a systematic account of the molecular regulatory effects of small non-coding FenSr3 in *B. amyloliquefaciens* LPB-18, especially fengycin biosynthesis. Production of fengycin, biofilm formation, and sporulation were greatly improved in the strain of LPB-18N. The precursor synthesis of fengycin was influenced by FenSr3, as shown by comparative sequencing studying, which acted to promote amino acid fatty acid biosynthesis and central carbon metabolism. Moreover, the biofilm formation and sporulation also showed a remarkable difference in *B. amyloliquefaciens* LPB-18N. The novel non-coding small RNA FenSr3 was identified in * B. amyloliquefaciens* LPB-18, and it was involved in fengycin producing and stress resistance. Thus, the results of this study will contribute novel dimensions to the regulation mechanism of metabolite biosynthesis and the optimization of crucial metabolites of *B. amyloliquefaciens.*

##  Supplemental Information

10.7717/peerj.15236/supp-1 Supplemental Information 1 The evolutionary footprint of sRNA FenSr3

10.7717/peerj.15236/supp-2 Supplemental Information 2 The raw data of fengycin production in different period

10.7717/peerj.15236/supp-3 Supplemental Information 3 The raw data of biomass

10.7717/peerj.15236/supp-4 Supplemental Information 4 Pathway enrichment analysis

10.7717/peerj.15236/supp-5 Supplemental Information 5 The raw data of Figure 5A

10.7717/peerj.15236/supp-6 Supplemental Information 6 The fengycin production of different type Bacillus amyloliquefaciens Liquid chromatography was used to detect Fengycin, and according to the comparison with a standard sample, it was found that peaks 1-6 corresponded to Fengycin products. The peak area can represent the substance content, and in strain LPB-18N, the peak area was the largest. Compared with the wild-type strain LPB-18 and the overexpression strain LPB-18P, the latter had almost no peak area, indicating that it did not produce Fengycin.

10.7717/peerj.15236/supp-7 Supplemental Information 7 Transcriptome sequencing information The complete routing group information.

10.7717/peerj.15236/supp-8 Supplemental Information 8 The regulatory pathway of fengycin and biofilm formation

## Additional Information and Declarations

### Data Availability

The following information was supplied regarding data availability: The data is available at NCBI Bioproject: PRJNA841238.
