# Supplementary figures and images for: Identification and functional analysis of non-coding regulatory small RNA FenSr3 in Bacillus amyloliquefaciens LPB-18
Source: PeerJ. 2023 May 15;11:e15236. doi: 10.7717/peerj.15236 (PMC10194069; doi:10.7717/peerj.15236)

Supplementary

The evolutionary footprint of sRNA FenSr3

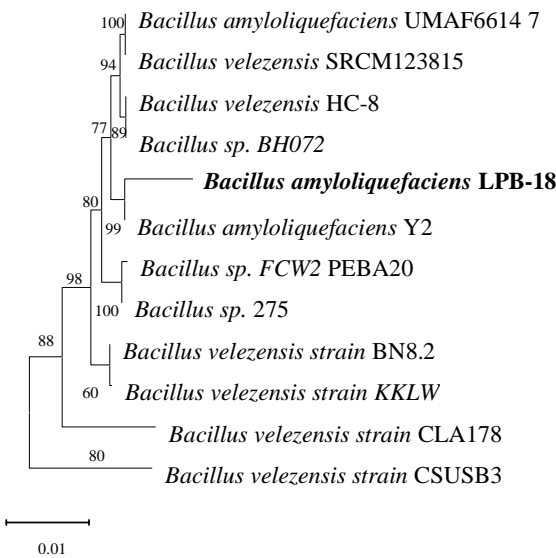

Supplement: Supplemental Information 1 [file peerj-11-15236-s001.pdf]
