# Supplementary material for: Identification and functional analysis of non-coding regulatory small RNA FenSr3 in Bacillus amyloliquefaciens LPB-18
Source: PeerJ. 2023 May 15;11:e15236. doi: 10.7717/peerj.15236 (PMC10194069; doi:10.7717/peerj.15236)
Supplement: Supplemental Information 4 [file peerj-11-15236-s004.zip › KO/CK-vs-T1_map/map00010.html]

KEGG PATHWAY: Glycolysis / Gluconeogenesis - Reference pathway


|  |  |
| --- | --- |
| **Glycolysis / Gluconeogenesis - Reference pathway** |  |

[
Pathway menu
| Organism menu
| Pathway entry
| Show description
| User data mapping
]

|  |
| --- |
| Glycolysis is the process of converting glucose into pyruvate and generating small amounts of ATP (energy) and NADH (reducing power). It is a central pathway that produces important precursor metabolites: six-carbon compounds of glucose-6P and fructose-6P and three-carbon compounds of glycerone-P, glyceraldehyde-3P, glycerate-3P, phosphoenolpyruvate, and pyruvate [MD:M00001]. Acetyl-CoA, another important precursor metabolite, is produced by oxidative decarboxylation of pyruvate [MD:M00307]. When the enzyme genes of this pathway are examined in completely sequenced genomes, the reaction steps of three-carbon compounds from glycerone-P to pyruvate form a conserved core module [MD:M00002], which is found in almost all organisms and which sometimes contains operon structures in bacterial genomes. Gluconeogenesis is a synthesis pathway of glucose from noncarbohydrate precursors. It is essentially a reversal of glycolysis with minor variations of alternative paths [MD:M00003]. |

|  |  |  |
| --- | --- | --- |
| Reference pathway | 184% 150% 122% 100% 82% 67% 55% | 图片下载 |
