# Supplementary material for: Identification and functional analysis of non-coding regulatory small RNA FenSr3 in Bacillus amyloliquefaciens LPB-18
Source: PeerJ. 2023 May 15;11:e15236. doi: 10.7717/peerj.15236 (PMC10194069; doi:10.7717/peerj.15236)
Supplement: Supplemental Information 4 [file peerj-11-15236-s004.zip › KO/CK-vs-T1_map/map00120.html]

KEGG PATHWAY: Primary bile acid biosynthesis - Reference pathway


|  |  |
| --- | --- |
| **Primary bile acid biosynthesis - Reference pathway** |  |

[
Pathway menu
| Organism menu
| Pathway entry
| Show description
| User data mapping
]

|  |
| --- |
| Bile acids are steroid carboxylic acids derived from cholesterol in vertebrates. The primary bile acids, cholic acid and chenodeoxycholic acid, are synthesized in the liver and conjugated with taurine or glycine before secretion via bile into the intestine. The conversion from cholesterol to cholic and chenodeoxycholic acids involves four steps: 1) the initiation of synthesis by 7alpha-hydroxylation of sterol precursors, 2) further modifications to the ring structures, 3) side-chain oxidation and shortening (cleavage) by three carbons, and 4) conjugation of the bile acid with taurine or glycine. |

|  |  |  |
| --- | --- | --- |
| Reference pathway | 184% 150% 122% 100% 82% 67% 55% | 图片下载 |
