# Supplementary material for: Identification and functional analysis of non-coding regulatory small RNA FenSr3 in Bacillus amyloliquefaciens LPB-18
Source: PeerJ. 2023 May 15;11:e15236. doi: 10.7717/peerj.15236 (PMC10194069; doi:10.7717/peerj.15236)
Supplement: Supplemental Information 4 [file peerj-11-15236-s004.zip › KO/CK-vs-T1_map/map00121.html]

KEGG PATHWAY: Secondary bile acid biosynthesis - Reference pathway


|  |  |
| --- | --- |
| **Secondary bile acid biosynthesis - Reference pathway** |  |

[
Pathway menu
| Organism menu
| Pathway entry
| Show description
| User data mapping
]

|  |
| --- |
| The secondary bile acids are derived from the primary bile acids by the enzymatic action of intestinal bacteria through the process of deconjugation and dehydroxylation. The secondary bile acids in humans include deoxycholic acid and lithocholic acid, formed from the 7alpha-dehydroxylation of cholic acid and chenodeoxycholic acid, respectively. |

|  |  |  |
| --- | --- | --- |
| Reference pathway | 184% 150% 122% 100% 82% 67% 55% | 图片下载 |
