# Supplementary material for: Identification and functional analysis of non-coding regulatory small RNA FenSr3 in Bacillus amyloliquefaciens LPB-18
Source: PeerJ. 2023 May 15;11:e15236. doi: 10.7717/peerj.15236 (PMC10194069; doi:10.7717/peerj.15236)
Supplement: Supplemental Information 4 [file peerj-11-15236-s004.zip › KO/CK-vs-T1_map/map00140.html]

KEGG PATHWAY: Steroid hormone biosynthesis - Reference pathway


|  |  |
| --- | --- |
| **Steroid hormone biosynthesis - Reference pathway** |  |

[
Pathway menu
| Organism menu
| Pathway entry
| Show description
| User data mapping
]

|  |
| --- |
| Steroid hormones derived from cholesterol are a class of biologically active compounds in vertebrates. The cholesterol side-chain cleavage enzyme CYP11A1 catalyzes conversion of cholesterol, a C27 compound, to the first C21 steroid, pregnenolone, which is converted by a bifunctional enzyme complex to the gestagen hormone, progesterone [MD:M00107]. Pregnenolone and progesterone are the starting materials for the three groups of steroids: C21 steroids of glucocorticoids and mineralocorticoids, C19 steroids of androgens, and C18 steroids of estrogens. (i) Progesterone is converted by hydroxylations at carbons 21 and 11 to corticosterone, which is further modified by hydroxylation and oxydoreduction at carbon 18 to yield aldosterone, a mineralcorticoid [MD:M00108]. Cortisol, the main glucocorticoid, is formed from 17alpha-hydroxyprogesterone with 11-deoxycortisol as an intermediate [MD:M00109]. (ii) Male hormone testosterone is formed from pregnenolone by two pathways, delta5 pathway via dehydroepiandrosterone and delta4 pathway via androstenedione [MD:M00110]. The enzyme CYP17A1 is responsible for the 17,20 lyase and 17alpha-hydroxylase activities in respective pathways. (iii) Female hormones estrone and estradiol are formed from testosterone and 4-androstene-3,17-dione by oxidative removal of the C19 methyl group and subsequent aromatization of ring A [MD:M00111]. In addition to these three groups, recent studies show that there is another group, termed neurosteroids, synthesized in the brain rather than the peripheral endocrine gland. |

|  |  |  |
| --- | --- | --- |
| Reference pathway | 184% 150% 122% 100% 82% 67% 55% | 图片下载 |
