# Supplementary material for: Identification and functional analysis of non-coding regulatory small RNA FenSr3 in Bacillus amyloliquefaciens LPB-18
Source: PeerJ. 2023 May 15;11:e15236. doi: 10.7717/peerj.15236 (PMC10194069; doi:10.7717/peerj.15236)
Supplement: Supplemental Information 4 [file peerj-11-15236-s004.zip › KO/CK-vs-T1_map/map00195.html]

KEGG PATHWAY: Photosynthesis - Reference pathway


|  |  |
| --- | --- |
| **Photosynthesis - Reference pathway** |  |

[
Pathway menu
| Organism menu
| Pathway entry
| Show description
| User data mapping
]

|  |
| --- |
| Photosynthesis in green plants and specialized bacteria is the process of utilizing light energy to synthesize organic compounds from carbon dioxide and water. It consists of the light dependent part (light reaction) and the light independent part (dark reaction, carbon fixation). The light reaction takes place in thylakoid, a membrane-bound compartment inside chloroplasts and cyanobacteria. The light energy is used by photosystems I and II to generate proton motive force and reducing power (NADPH or NADH). The proton motive force is used by ATP synthase to generate ATP, essentially in the same way as the mitochondrial respiratory chain. The supplies of ATP and NAD(P)H are then used to fix carbon dioxide. |

|  |  |  |
| --- | --- | --- |
| Reference pathway | 184% 150% 122% 100% 82% 67% 55% | 图片下载 |
