# Supplementary material for: Identification and functional analysis of non-coding regulatory small RNA FenSr3 in Bacillus amyloliquefaciens LPB-18
Source: PeerJ. 2023 May 15;11:e15236. doi: 10.7717/peerj.15236 (PMC10194069; doi:10.7717/peerj.15236)
Supplement: Supplemental Information 4 [file peerj-11-15236-s004.zip › KO/CK-vs-T1_map/map00253.html]

KEGG PATHWAY: Tetracycline biosynthesis - Reference pathway


|  |  |
| --- | --- |
| **Tetracycline biosynthesis - Reference pathway** |  |

[
Pathway menu
| Organism menu
| Pathway entry
| Show description
| User data mapping
]

|  |
| --- |
| Tetracyclines are aromatic polyketide antibiotics produced by Streptomyces species via type II polyketide synthases (PKSs). Tetracyclines contain a linear tetracyclic skeleton, which is formed from a malonamate starter unit and malonyl-CoA extender units through a common polyketide pathway [MD:M00778]. This diagram shows biosynthesis of naturally occurring tetracyclines (tetracycline, oxytetracycline and chlortetracycline) via a common intermediate anhydrotetracycline [MD:M00780 M00823]. |

|  |  |  |
| --- | --- | --- |
| Reference pathway | 184% 150% 122% 100% 82% 67% 55% | 图片下载 |
