# Supplementary material for: Identification and functional analysis of non-coding regulatory small RNA FenSr3 in Bacillus amyloliquefaciens LPB-18
Source: PeerJ. 2023 May 15;11:e15236. doi: 10.7717/peerj.15236 (PMC10194069; doi:10.7717/peerj.15236)
Supplement: Supplemental Information 4 [file peerj-11-15236-s004.zip › KO/CK-vs-T1_map/map00261.html]

KEGG PATHWAY: Monobactam biosynthesis - Reference pathway


|  |  |
| --- | --- |
| **Monobactam biosynthesis - Reference pathway** |  |

[
Pathway menu
| Organism menu
| Pathway entry
| Show description
| User data mapping
]

|  |
| --- |
| Monobactams are beta-lactam antibiotics containing a monocyclic beta-lactam nucleus, which is structurally different from penicillin and cephalosporin core structures with another fused ring. This diagram shows biosynthesis of nocardicin A, a naturally occurring monobactam, via the pentapeptide formed by condensation of L-4-hydroxyphenylglycine (L-pHPG), L-arginine and L-serine [MD:M00736]. Other naturally occurring monobactams are also shown but the biosynthetic pathway is not yet fully characterized. Sulfazecin and other 3-aminomonobactamic acid derivatives are derived from serine or threonine, and tabtoxinine-beta-lactam is a phytotoxin. Aztreonam, a synthetic monobactam originally isolated as SQ 26,180 from Chromobacterium violaceum, is the first clinically used monobactam. |

|  |  |  |
| --- | --- | --- |
| Reference pathway | 184% 150% 122% 100% 82% 67% 55% | 图片下载 |
