# Supplementary material for: Identification and functional analysis of non-coding regulatory small RNA FenSr3 in Bacillus amyloliquefaciens LPB-18
Source: PeerJ. 2023 May 15;11:e15236. doi: 10.7717/peerj.15236 (PMC10194069; doi:10.7717/peerj.15236)
Supplement: Supplemental Information 4 [file peerj-11-15236-s004.zip › KO/CK-vs-T1_map/map00270.html]

KEGG PATHWAY: Cysteine and methionine metabolism - Reference pathway


|  |  |
| --- | --- |
| **Cysteine and methionine metabolism - Reference pathway** |  |

[
Pathway menu
| Organism menu
| Pathway entry
| Show description
| User data mapping
]

|  |
| --- |
| Cysteine and methionine are sulfur-containing amino acids. Cysteine is synthesized from serine through different pathways in different organism groups. In bacteria and plants, cysteine is converted from serine (via acetylserine) by transfer of hydrogen sulfide [MD:M00021]. In animals, methionine-derived homocysteine is used as sulfur source and its condensation product with serine (cystathionine) is converted to cysteine [MD:M00338]. Cysteine is metabolized to pyruvate in multiple routes. Methionine is an essential amino acid, which animals cannot synthesize. In bacteria and plants, methionine is synthesized from aspartate [MD:M00017]. S-Adenosylmethionine (SAM), synthesized from methionine and ATP, is a methyl group donor in many important transfer reactions including DNA methylation for regulation of gene expression. SAM may also be used to regenerate methionine in the methionine salvage pathway [MD:M00034]. |

|  |  |  |
| --- | --- | --- |
| Reference pathway | 184% 150% 122% 100% 82% 67% 55% | 图片下载 |
