# Supplementary material for: Identification and functional analysis of non-coding regulatory small RNA FenSr3 in Bacillus amyloliquefaciens LPB-18
Source: PeerJ. 2023 May 15;11:e15236. doi: 10.7717/peerj.15236 (PMC10194069; doi:10.7717/peerj.15236)
Supplement: Supplemental Information 4 [file peerj-11-15236-s004.zip › KO/CK-vs-T1_map/map00311.html]

KEGG PATHWAY: Penicillin and cephalosporin biosynthesis - Reference pathway


|  |  |
| --- | --- |
| **Penicillin and cephalosporin biosynthesis - Reference pathway** |  |

[
Pathway menu
| Organism menu
| Pathway entry
| Show description
| User data mapping
]

|  |
| --- |
| Penicillins (Penams) and cephalosporins (cephems) are beta-lactam antibiotics containing 6-aminopenicillanate (6-APA) and 7-aminocephalosporanate (7-ACA) nuclei, respectively. 6-APA and 7-ACA are key intermediates for a variety of semisynthetic penicillin and cephalosporin derivatives. Penicillins are produced only by fungi, while cephalosporins (including cephamycins) are produced by fungi and bacteria. Both antibiotics are synthesized from L-2-aminoadipate, L-cysteine and L-valine through a common pathway. It starts with the condensation of these three amino acids by the non-ribosomal peptide synthetase to form the tripeptide delta-(L-2-aminoadipyl)-L-cysteinyl-D-valine (ACV). The linear ACV tripeptide is then converted to bicyclic isopenicillin N by isopenicillin N synthase, in which the beta-lactam ring is formed. Isopenicillin N is the branch point of penicillin [MD:M00672] and cephalosporin [MD:M00673] pathways. |

|  |  |  |
| --- | --- | --- |
| Reference pathway | 184% 150% 122% 100% 82% 67% 55% | 图片下载 |
