# Supplementary material for: Identification and functional analysis of non-coding regulatory small RNA FenSr3 in Bacillus amyloliquefaciens LPB-18
Source: PeerJ. 2023 May 15;11:e15236. doi: 10.7717/peerj.15236 (PMC10194069; doi:10.7717/peerj.15236)
Supplement: Supplemental Information 4 [file peerj-11-15236-s004.zip › KO/CK-vs-T1_map/map00332.html]

KEGG PATHWAY: Carbapenem biosynthesis - Reference pathway


|  |  |
| --- | --- |
| **Carbapenem biosynthesis - Reference pathway** |  |

[
Pathway menu
| Organism menu
| Pathway entry
| Show description
| User data mapping
]

|  |
| --- |
| Carbapenems are broad-spectrum beta-lactam antibiotics, which are often considered as the antibiotics of last resort. A naturally occurring carbapenem, thienamycin, was first discovered in Streptomyces cattleya. This diagram shows how a simple carbapenem, carbapenem-3-carboxylate, is synthesized from malonyl-CoA and pyrroline-5-carboxylate [MD:M00675]. For structurally complex carbapenems, such as thienamycin, olivanic acid, epi-thienamycin and carbapenems of the OA-6129 group, uncertainty remains about the mechanism and timing for the inversion of the C-5 carbapenam bridgehead, the desaturation of the C-2/C-3 bond and the attachment of the C-2 and C-6 side chains. |

|  |  |  |
| --- | --- | --- |
| Reference pathway | 184% 150% 122% 100% 82% 67% 55% | 图片下载 |
