# Supplementary material for: Identification and functional analysis of non-coding regulatory small RNA FenSr3 in Bacillus amyloliquefaciens LPB-18
Source: PeerJ. 2023 May 15;11:e15236. doi: 10.7717/peerj.15236 (PMC10194069; doi:10.7717/peerj.15236)
Supplement: Supplemental Information 4 [file peerj-11-15236-s004.zip › KO/CK-vs-T1_map/map00333.html]

KEGG PATHWAY: Prodigiosin biosynthesis - Reference pathway


|  |  |
| --- | --- |
| **Prodigiosin biosynthesis - Reference pathway** |  |

[
Pathway menu
| Organism menu
| Pathway entry
| Show description
| User data mapping
]

|  |
| --- |
| Prodiginines are red-pigmented natural antibiotics that are produced as secondary metabolites and have received renewed attention because of potential clinical interests. In Serratia sp. or actinomycetes such as Streptomyces coelicolor, the tripyrrole molecules of prodigiosin and undecylprodigiosin are formed by the condensation of 4-methoxy-2,2'-bipyrrole-5-carbaldehyde and either 2-methyl-3-n-amyl-pyrrole [MD:M00837] or 2-undecylpyrrole [MD:M00838]. The Serratia pig gene cluster and the Streptomyces red gene cluster responsible for these biosynthesis pathways have been identified. |

|  |  |  |
| --- | --- | --- |
| Reference pathway | 184% 150% 122% 100% 82% 67% 55% | 图片下载 |
