# Supplementary material for: Identification and functional analysis of non-coding regulatory small RNA FenSr3 in Bacillus amyloliquefaciens LPB-18
Source: PeerJ. 2023 May 15;11:e15236. doi: 10.7717/peerj.15236 (PMC10194069; doi:10.7717/peerj.15236)
Supplement: Supplemental Information 4 [file peerj-11-15236-s004.zip › KO/CK-vs-T1_map/map00402.html]

KEGG PATHWAY: Benzoxazinoid biosynthesis - Reference pathway


|  |  |
| --- | --- |
| **Benzoxazinoid biosynthesis - Reference pathway** |  |

[
Pathway menu
| Organism menu
| Pathway entry
| Show description
| User data mapping
]

|  |
| --- |
| Benzoxazinoids (hydroxamic acids) are plant secondary metabolites that serve as important factors for host resistance against microbial pathogens and insects and for allelopathic effects. They are found in grass family and some eudicot families. The predominant benzoxazinoids are DIBOA and its 7-methoxy derivative DIMBOA, which are stored as glucosides in vacuoles. In maize, benzoxazinoid biosynthesis branches off from tryptophan biosynthesis at indole-3-glycerol phosphate, which is converted to indole by indole-3-glycerol phosphate lyase, BX1. Subsequently four cytochrome P450 monooxygenases (BX2-BX5) catalyze the introduction of four oxygen atoms into the indole moiety, yielding DIBOA. After glucosylation by UDP-glucosyltransferase (BX8/BX9), the glucoside is further modified by hydroxylation and O-methylation at C-7 to form DIMBOA-glucoside. |

|  |  |  |
| --- | --- | --- |
| Reference pathway | 184% 150% 122% 100% 82% 67% 55% | 图片下载 |
