# Supplementary material for: Identification and functional analysis of non-coding regulatory small RNA FenSr3 in Bacillus amyloliquefaciens LPB-18
Source: PeerJ. 2023 May 15;11:e15236. doi: 10.7717/peerj.15236 (PMC10194069; doi:10.7717/peerj.15236)
Supplement: Supplemental Information 4 [file peerj-11-15236-s004.zip › KO/CK-vs-T1_map/map00403.html]

KEGG PATHWAY: Indole diterpene alkaloid biosynthesis - Reference pathway


|  |  |
| --- | --- |
| **Indole diterpene alkaloid biosynthesis - Reference pathway** |  |

[
Pathway menu
| Organism menu
| Pathway entry
| Show description
| User data mapping
]

|  |
| --- |
| Indole diterpene alkaloids are a group of meroterpenoids consisting of a partial diterpene structure with four isoprene units and an indole moiety. The common cyclic diterpene skeleton is derived from geranylgeranyl diphosphate (GGPP) and an indole moiety is derived from tryptophan or a tryptophan precursor. They are combined in the initial step of biosynthetic reactions that generate paspaline, the first stable and less modified form required for other forms. Most indole diterpene alkaloids have potent mammalian tremorgenic activities, such as ryegrass staggers in livestock, and some have anti-insect properties. This map shows examples of indole diterpene alkaloids isolated from filamentous fungi. |

|  |  |  |
| --- | --- | --- |
| Reference pathway | 184% 150% 122% 100% 82% 67% 55% | 图片下载 |
