# Supplementary material for: Identification and functional analysis of non-coding regulatory small RNA FenSr3 in Bacillus amyloliquefaciens LPB-18
Source: PeerJ. 2023 May 15;11:e15236. doi: 10.7717/peerj.15236 (PMC10194069; doi:10.7717/peerj.15236)
Supplement: Supplemental Information 4 [file peerj-11-15236-s004.zip › KO/CK-vs-T1_map/map00440.html]

KEGG PATHWAY: Phosphonate and phosphinate metabolism - Reference pathway


|  |  |
| --- | --- |
| **Phosphonate and phosphinate metabolism - Reference pathway** |  |

[
Pathway menu
| Organism menu
| Pathway entry
| Show description
| User data mapping
]

|  |
| --- |
| Natural products containing carbon-phosphorous bonds, so-called C-P compounds, are derivatives of phosphonate and phosphinate with substitution of alkyl group for hydrogen of phosphorus-hydrogen bonds. C-P compounds have been found in many organisms, but only protists and bacteria, mostly Actinobacteria, have biosynthetic capacity. A common reaction in the biosynthetic pathway is C-P bond forming reaction from phosphoenolpyruvate (PEP) to phosphonopyruvate (PnPy) catalyzed by PEP phosphomutase. 2-Aminoethylphosphonate (AEP) is the most abundant C-P compound in the natural world. AEP derivatives include phosphonoprotein, phosphonoglycan, and phosphonolipid. Other known C-P compounds are bioactive substances used in medicine (antibiotics) and agriculture (herbicide) such as fosfomycin, FR-33289, rhizocticin, and bialaphos. |

|  |  |  |
| --- | --- | --- |
| Reference pathway | 184% 150% 122% 100% 82% 67% 55% | 图片下载 |
