# Supplementary material for: Identification and functional analysis of non-coding regulatory small RNA FenSr3 in Bacillus amyloliquefaciens LPB-18
Source: PeerJ. 2023 May 15;11:e15236. doi: 10.7717/peerj.15236 (PMC10194069; doi:10.7717/peerj.15236)
Supplement: Supplemental Information 4 [file peerj-11-15236-s004.zip › KO/CK-vs-T1_map/map00510.html]

KEGG PATHWAY: N-Glycan biosynthesis - Reference pathway


|  |  |
| --- | --- |
| **N-Glycan biosynthesis - Reference pathway** |  |

[
Pathway menu
| Organism menu
| Pathway entry
| Show description
| User data mapping
]

|  |
| --- |
| N-glycans or asparagine-linked glycans are major constituents of glycoproteins in eukaryotes. N-glycans are covalently attached to asparagine with the consensus sequence of Asn-X-Ser/Thr by an N-glycosidic bond, GlcNAc b1- Asn. Biosynthesis of N-glycans begins on the cytoplasmic face of the ER membrane with the transferase reaction of UDP-GlcNAc and the lipid-like precursor P-Dol (dolichol phosphate) to generate GlcNAc a1- PP-Dol. After sequential addition of monosaccharides by ALG glycosyltransferases [MD:M00055], the N-glycan precursor is attached by the OST (oligosaccharyltransferase) complex to the polypeptide chain that is being synthesized and translocated through the ER membrane. The protein-bound N-glycan precursor is subsequently trimmed, extended, and modified in the ER and Golgi by a complex series of reactions catalyzed by membrane-bound glycosidases and glycosyltransferases. N-glycans thus synthesized are classified into three types: high-mannose type, complex type, and hybrid type. Defects in N-glycan biosynthesis lead to a variety of human diseases known as congenital disorders of glycosylation [DS:H00118 H00119]. |

|  |  |  |
| --- | --- | --- |
| Reference pathway | 184% 150% 122% 100% 82% 67% 55% | 图片下载 |
