# Supplementary material for: Identification and functional analysis of non-coding regulatory small RNA FenSr3 in Bacillus amyloliquefaciens LPB-18
Source: PeerJ. 2023 May 15;11:e15236. doi: 10.7717/peerj.15236 (PMC10194069; doi:10.7717/peerj.15236)
Supplement: Supplemental Information 4 [file peerj-11-15236-s004.zip › KO/CK-vs-T1_map/map00512.html]

KEGG PATHWAY: Mucin type O-glycan biosynthesis - Reference pathway


|  |  |
| --- | --- |
| **Mucin type O-glycan biosynthesis - Reference pathway** |  |

[
Pathway menu
| Organism menu
| Pathway entry
| Show description
| User data mapping
]

|  |
| --- |
| O-glycans are a class of glycans that modify serine or threonine residues of proteins. Biosynthesis of O-glycans starts from the transfer of N-acetylgalactosamine (GalNAc) to serine or threonine. The first GalNAc may be extended with sugars including galactose, N-acetylglucosamine, fucose, or sialic acid, but not mannose, glucose, or xylose. Depending on the sugars added, there are four common O-glycan core structures, cores 1 through 4, and an additional four, cores 5 though 8. Mucins are highly O-glycosylated glycoproteins ubiquitous in mucous secretions on cell surfaces and in body fluids. Mucin O-glycans can be branched, and many sugars or groups of sugars are antigenic. Important modifications of mucin O-glycans include O-acetylation of sialic acid and O-sulfation of galactose and N-acetylglucosamine. |

|  |  |  |
| --- | --- | --- |
| Reference pathway | 184% 150% 122% 100% 82% 67% 55% | 图片下载 |
