# Supplementary material for: Identification and functional analysis of non-coding regulatory small RNA FenSr3 in Bacillus amyloliquefaciens LPB-18
Source: PeerJ. 2023 May 15;11:e15236. doi: 10.7717/peerj.15236 (PMC10194069; doi:10.7717/peerj.15236)
Supplement: Supplemental Information 4 [file peerj-11-15236-s004.zip › KO/CK-vs-T1_map/map00514.html]

KEGG PATHWAY: Other types of O-glycan biosynthesis - Reference pathway


|  |  |
| --- | --- |
| **Other types of O-glycan biosynthesis - Reference pathway** |  |

[
Pathway menu
| Organism menu
| Pathway entry
| Show description
| User data mapping
]

|  |
| --- |
| O-linked glycosylation is the attachment of monosaccharides to the hydroxyl groups of amino acids, mostly serine and threonine, and is found in eukaryotes, archaea and bacteria. O-glycans exhibit diverse types of modifications where the innermost monosaccharide is N-acetylgalactosamine (map00512), mannose in mammals (map00515), and others (this map) including N-acetylglucosamine, fucose, glucose, galactose, mannose in yeast and arabinose in plants. |

|  |  |  |
| --- | --- | --- |
| Reference pathway | 184% 150% 122% 100% 82% 67% 55% | 图片下载 |
