# Supplementary material for: Identification and functional analysis of non-coding regulatory small RNA FenSr3 in Bacillus amyloliquefaciens LPB-18
Source: PeerJ. 2023 May 15;11:e15236. doi: 10.7717/peerj.15236 (PMC10194069; doi:10.7717/peerj.15236)
Supplement: Supplemental Information 4 [file peerj-11-15236-s004.zip › KO/CK-vs-T1_map/map00515.html]

KEGG PATHWAY: Mannose type O-glycan biosynthesis - Reference pathway


|  |  |
| --- | --- |
| **Mannose type O-glycan biosynthesis - Reference pathway** |  |

[
Pathway menu
| Organism menu
| Pathway entry
| Show description
| User data mapping
]

|  |
| --- |
| Biosynthesis of mammalian O-mannosyl glycans is initiated by the transfer of mannose from mannose-P-Dol to serine or threonine residue, followed by extensions with N-acetylglucosamine (GlcNAc) and galactose (Gal) to generate core M1, M2 and M3 glycans. Core M1 and M2 glycans can then be further attached by fucose residues, sialic acid terminals and sulfatded glucuroinc acid terminals. Core M3 glycan is involved in the synthesis of alpha-dystroglycan, a heavily glycosylated protein found in muscle and brain tissues. Core M3 glycan contains a tandem repeat of ribitol 5-phosphate (Rbo5P) and -alpha3-GlcA-beta3-Xyl- repeating structures. Defects of genes encoding core glycans and modified core M3 glycans are associated with various congenital diseases, such as muscular dystrophies caused by reduced O-mannosylation of alpha-dystroglycan in skeletal muscles [DS:H00120]. |

|  |  |  |
| --- | --- | --- |
| Reference pathway | 184% 150% 122% 100% 82% 67% 55% | 图片下载 |
