# Supplementary material for: Identification and functional analysis of non-coding regulatory small RNA FenSr3 in Bacillus amyloliquefaciens LPB-18
Source: PeerJ. 2023 May 15;11:e15236. doi: 10.7717/peerj.15236 (PMC10194069; doi:10.7717/peerj.15236)
Supplement: Supplemental Information 4 [file peerj-11-15236-s004.zip › KO/CK-vs-T1_map/map00521.html]

KEGG PATHWAY: Streptomycin biosynthesis - Reference pathway


|  |  |
| --- | --- |
| **Streptomycin biosynthesis - Reference pathway** |  |

[
Pathway menu
| Organism menu
| Pathway entry
| Show description
| User data mapping
]

|  |
| --- |
| Streptomycin is an aminocyclitol-aminoglycoside antibiotic produced by Streptomyces griseus. Streptomycin consists of aminocyclitol (streptidine), 6-deoxyhexose (streptose), and N-methyl-L-glucosamine moieties, which are formed by independent biosynthetic pathways. All of the moieties are derived from D-glucose. The streptidine moiety is synthesized via myo-inositol, which is then oxidized at C-1 and transaminated to give scyllo-inosamine. After phosphorylation, the compound is transamidinated by arginine. The same procedure is repeated at the C-3 position. The streptose moiety is made from D-glucose via a dTDP-glucose pathway. The exact biosynthetic route for the N-methyl-L-glucosamine moiety is unknown, though the biosynthetic gene cluster have been proposed. |

|  |  |  |
| --- | --- | --- |
| Reference pathway | 184% 150% 122% 100% 82% 67% 55% | 图片下载 |
