# Supplementary material for: Identification and functional analysis of non-coding regulatory small RNA FenSr3 in Bacillus amyloliquefaciens LPB-18
Source: PeerJ. 2023 May 15;11:e15236. doi: 10.7717/peerj.15236 (PMC10194069; doi:10.7717/peerj.15236)
Supplement: Supplemental Information 4 [file peerj-11-15236-s004.zip › KO/CK-vs-T1_map/map00532.html]

KEGG PATHWAY: Glycosaminoglycan biosynthesis - chondroitin sulfate / dermatan sulfate - Reference pathway


|  |  |
| --- | --- |
| **Glycosaminoglycan biosynthesis - chondroitin sulfate / dermatan sulfate - Reference pathway** |  |

[
Pathway menu
| Organism menu
| Pathway entry
| Show description
| User data mapping
]

|  |
| --- |
| Glycosaminoglycans (GAGs) are linear polysaccharide chains consisting of repeating disaccharide units and form proteglycans by covalently attaching to their core proteins. Chondroitin sulfate (CS) is a glycosaminoglycan with the disaccharide unit of beta-D-galactosamine (GalNAc) and beta-D-glucuronic acid (GlcA), and often modified with ester-linked sulfate at certain positions. Dermatan sulfate (DS) is a modified form of CS, in which a portion of D-glucuronate residues is epimerized to L-iduronates (IdoA). CS and DS are linked to serine residues in core proteins via a linkage tetrasaccharide formed by the transfer of xylose and three more residues [MD:M00057]. The assembly process of CS is initiated by transferring GalNAc residue to the linkage tetrasaccharide. The polymerization is catalyzed by bifunctional enzymes (chondroitin synthases) possessing both beta 1,3 glucuronosyltransferase and beta 1,4 N-acetylgalactosaminyltransferase activities [MD:M00058]. Chondroitin polymerization also requires the action of the chondroitin polymerizing factor. There are various O-sulfation patterns in CS and DS, where 4-O sulfation and 6-O sulfation of GalNAc and 2-O-sulfation of the uronic acids (GlcA / IdoA) are mainly found. |

|  |  |  |
| --- | --- | --- |
| Reference pathway | 184% 150% 122% 100% 82% 67% 55% | 图片下载 |
