# Supplementary material for: Identification and functional analysis of non-coding regulatory small RNA FenSr3 in Bacillus amyloliquefaciens LPB-18
Source: PeerJ. 2023 May 15;11:e15236. doi: 10.7717/peerj.15236 (PMC10194069; doi:10.7717/peerj.15236)
Supplement: Supplemental Information 4 [file peerj-11-15236-s004.zip › KO/CK-vs-T1_map/map00534.html]

KEGG PATHWAY: Glycosaminoglycan biosynthesis - heparan sulfate / heparin - Reference pathway


|  |  |
| --- | --- |
| **Glycosaminoglycan biosynthesis - heparan sulfate / heparin - Reference pathway** |  |

[
Pathway menu
| Organism menu
| Pathway entry
| Show description
| User data mapping
]

|  |
| --- |
| Heparan sulfate (HS) and heparin (Hep) are glycosaminoglycans with repeating disaccharide units that consist of alternating residues of alpha-D-glucosamine (GlcN) and uronic acid, the latter being either beta-D-glucuronic acid (GlcA) or alpha-L-iduronic acid (IdoA). In these sugar residues, sulfation modification may be performed at various positions. Structural studies show that Hep possesses a higher degree of sulfation than HS. The biosynthesis of HS/Hep occurs with the addition of the first GlcNAc residue by EXTL3 glycosyltransferase after completion of tetrasaccharide linkage region attached to serine residue of a core protein. The chain polymeraization is then catalyzed by EXT1 and EXT2 transferases. As the chain polymerizes, HS/Hep undergoes a series of modification reactions including N-deacetylation, N-sulfation, epimerization, and subsequently O-sulfation. As final products of biosynthesis, HS is present in the form of hepran sulfate proteoglycan (HSPG) whereas Hep exists as a sugar chain without a core protein. The proteoglycan families with HS, as well as CS (chondroitin sulfate), DS (dermatan sulfate), and KS (keratan sulfate), are composed of two main types depending on the subcellular locations: cell membrane and extracellular matrix [BR:ko00535]. HS/Hep has been shown to bind to a variety of molecules, such as growth factors, chemokines, morphogens, and extracellular matrix components [BR:ko00536]. |

|  |  |  |
| --- | --- | --- |
| Reference pathway | 184% 150% 122% 100% 82% 67% 55% | 图片下载 |
