# Supplementary material for: Identification and functional analysis of non-coding regulatory small RNA FenSr3 in Bacillus amyloliquefaciens LPB-18
Source: PeerJ. 2023 May 15;11:e15236. doi: 10.7717/peerj.15236 (PMC10194069; doi:10.7717/peerj.15236)
Supplement: Supplemental Information 4 [file peerj-11-15236-s004.zip › KO/CK-vs-T1_map/map00550.html]

KEGG PATHWAY: Peptidoglycan biosynthesis - Reference pathway


|  |  |
| --- | --- |
| **Peptidoglycan biosynthesis - Reference pathway** |  |

[
Pathway menu
| Organism menu
| Pathway entry
| Show description
| User data mapping
]

|  |
| --- |
| Peptidoglycan is a macromolecule made of long aminosugar strands cross-linked by short peptides. It forms the cell wall in bacteria surrounding the cytoplasmic membrane. The glycan strands are typically comprised of repeating N-acetylglucosamine (GlcNAc) and N-acetylmuramic acid (MurNAc) disaccharides. Each MurNAc is linked to a peptide of three to five amino acid residues. Disaccharide subunits are first assembled on the cytoplasmic side of the bacterial membrane on a polyisoprenoid anchor (lipid I and II). Polymerization of disaccharide subunits by transglycosylases and cross-linking of glycan strands by transpeptidases occur on the other side of the membrane. Bacterial cell wall biosynthesis inhibitors form a major class of antibiotics. |

|  |  |  |
| --- | --- | --- |
| Reference pathway | 184% 150% 122% 100% 82% 67% 55% | 图片下载 |
