# Supplementary material for: Identification and functional analysis of non-coding regulatory small RNA FenSr3 in Bacillus amyloliquefaciens LPB-18
Source: PeerJ. 2023 May 15;11:e15236. doi: 10.7717/peerj.15236 (PMC10194069; doi:10.7717/peerj.15236)
Supplement: Supplemental Information 4 [file peerj-11-15236-s004.zip › KO/CK-vs-T1_map/map00563.html]

KEGG PATHWAY: Glycosylphosphatidylinositol (GPI)-anchor biosynthesis - Reference pathway


|  |  |
| --- | --- |
| **Glycosylphosphatidylinositol (GPI)-anchor biosynthesis - Reference pathway** |  |

[
Pathway menu
| Organism menu
| Pathway entry
| Show description
| User data mapping
]

|  |
| --- |
| Cell surface proteins can be attached to the cell membrane via the glycolipid structure called glycosylphosphatidylinositol (GPI) anchor. Hundreds of GPI-anchored proteins have been identified in many eukaryotes ranging from protozoa and fungi to mammals. All protein-linked GPI anchors share a common core structure, characterized by the substructure Man (a1-4) GlcN (a1-6) myo-inositol-1P-lipid. Biosynthesis of GPI anchors proceeds in three stages: (i) preassembly of a GPI precursor in the ER membrane, (ii) attachment of the GPI to the C-terminus of a newly synthesized protein in the lumen of the ER, and (iii) lipid remodeling and/or carbohydrate side-chain modifications in the ER and the Golgi. Defects of GPI anchor biosynthesis gene result in a genetic disorder, paroxysmal nocturnal hemoglobinuria. |

|  |  |  |
| --- | --- | --- |
| Reference pathway | 184% 150% 122% 100% 82% 67% 55% | 图片下载 |
