# Supplementary material for: Identification and functional analysis of non-coding regulatory small RNA FenSr3 in Bacillus amyloliquefaciens LPB-18
Source: PeerJ. 2023 May 15;11:e15236. doi: 10.7717/peerj.15236 (PMC10194069; doi:10.7717/peerj.15236)
Supplement: Supplemental Information 4 [file peerj-11-15236-s004.zip › KO/CK-vs-T1_map/map00571.html]

KEGG PATHWAY: Lipoarabinomannan (LAM) biosynthesis - Reference pathway


|  |  |
| --- | --- |
| **Lipoarabinomannan (LAM) biosynthesis - Reference pathway** |  |

[
Pathway menu
| Organism menu
| Pathway entry
| Show description
| User data mapping
]

|  |
| --- |
| Lipoarabinomannan (LAM), as well as structurally related lipomannan (LM) and phosphatidylinositol mannosides (PIMs), are major glycolipids found on the mycobacterial cell wall. LAM is synthesized from phosphatidylinositol (PI) and in a biochemical pathway of PI => PIMs => LM =>LAM. Structually, LAM and LM are an extension of PIMs containing an alpha1,6-linked mannan core with alpha1,2-monomannose side chains. The mannan core of LAM is in turn arabinosylated by a linear alpha1,5-linked Araf backbone, punctutated by alpha1,3-linked branching. LAM can be substituted by several capping motifs, which determine the ability of LAM to modulate the immune response through the interaction with different receptors containing C-type lectins (map04625). It has been shown that the immunomodulatory properties of LAM and related glycolipids contribute to the survival of Mycobacteriaum tuberculosis, the causative agent of tuberculosis (map05152). |

|  |  |  |
| --- | --- | --- |
| Reference pathway | 184% 150% 122% 100% 82% 67% 55% | 图片下载 |
