# Supplementary material for: Identification and functional analysis of non-coding regulatory small RNA FenSr3 in Bacillus amyloliquefaciens LPB-18
Source: PeerJ. 2023 May 15;11:e15236. doi: 10.7717/peerj.15236 (PMC10194069; doi:10.7717/peerj.15236)
Supplement: Supplemental Information 4 [file peerj-11-15236-s004.zip › KO/CK-vs-T1_map/map00621.html]

KEGG PATHWAY: Dioxin degradation - Reference pathway


|  |  |
| --- | --- |
| **Dioxin degradation - Reference pathway** |  |

[
Pathway menu
| Organism menu
| Pathway entry
| Show description
| User data mapping
]

|  |
| --- |
| Dioxins and dioxin-like compounds, including PCDDs, PCDFs and PCBs, are persistent organic pollutants (POPs) that are resistant to environmental degradation. However, POPs can be degraded by certain microorganisms with acquisition of a novel set of enzymes, such as biphenyl degradation by Paraburkholderia xenovorans LB400 involving four novel enzymes encoded in operon-like structures [MD:M00543]. DDT, another POP, and carbazol are also known to be degraded in similar pathways. |

|  |  |  |
| --- | --- | --- |
| Reference pathway | 184% 150% 122% 100% 82% 67% 55% | 图片下载 |
