# Supplementary material for: Identification and functional analysis of non-coding regulatory small RNA FenSr3 in Bacillus amyloliquefaciens LPB-18
Source: PeerJ. 2023 May 15;11:e15236. doi: 10.7717/peerj.15236 (PMC10194069; doi:10.7717/peerj.15236)

The fengycin production of different type *Bacillus amyloliquefaciens*

Bacillus amyloliquefaciens LPB-18

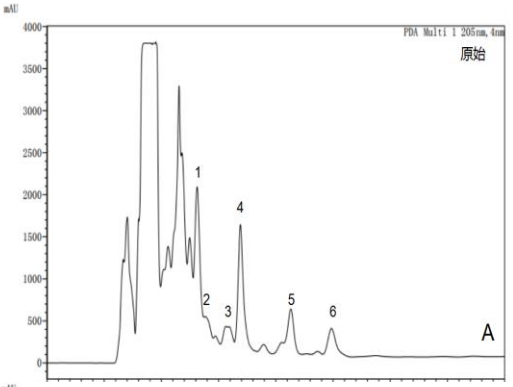

Bacillus amyloliquefaciens LPB-18N

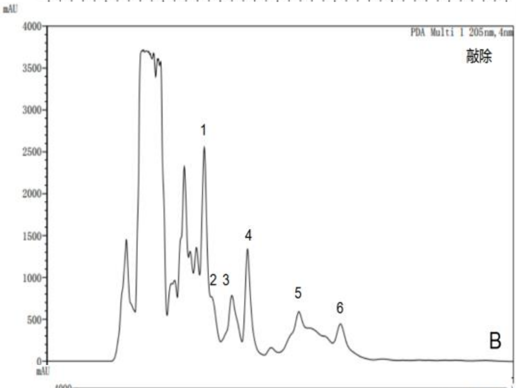

Bacillus amyloliquefaciens LPB-18P

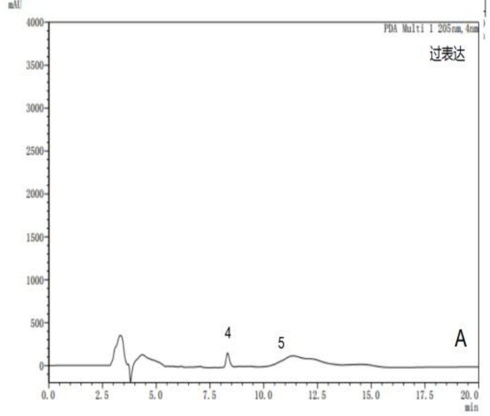

Supplement: Supplemental Information 6 — Liquid chromatography was used to detect Fengycin, and according to the comparison with a standard sample, it was found that peaks 1-6 corresponded to Fengycin products. The peak area can represent the substance content, and in strain LPB-18N, the peak area was the largest. Compared with the wild-type strain LPB-18 and the overexpression strain LPB-18P, the latter had almost no peak area, indicating that it did not produce Fengycin. [file peerj-11-15236-s006.pdf]
