# Supplementary material for: Identification and functional analysis of non-coding regulatory small RNA FenSr3 in Bacillus amyloliquefaciens LPB-18
Source: PeerJ. 2023 May 15;11:e15236. doi: 10.7717/peerj.15236 (PMC10194069; doi:10.7717/peerj.15236)
Supplement: Supplemental Information 7 — The complete routing group information. [file peerj-11-15236-s007.pdf]

Transcriptome sequencing information

(note: CK-1, CK-2, and CK-3 represent the transcriptome of wild-type *Bacillus amyloliquefaciens* LPB-18; T1-1, T2-2 and T3-3 represent the transcriptome of the deletion of sRNA FenSr3 in *Bacillus amyloliquefaciens* LPB-18N)

Table 1 Statistical table of base information before and after filtering

Sample	Before Filter					After Filter				
	Raw Data(bp)	Q20(%)	Q30(%)	N(%)	GC(%)	Clean Data(bp)	Q20(%)	Q30(%)	N(%)	GC(%)
CK-1	5007550800	4892200866 (97.7%)	4683425923 (93.53%)	9980 (0.0%)	2452255045 (48.97%)	4881061049	4772508217 (97.78%)	4571164431 (93.65%)	9721 (0.0%)	2386074245 (48.88%)
CK-2	4551865800	4460960707 (98.0%)	4286845307 (94.18%)	8877 (0.0%)	2231361454 (49.02%)	4422098148	4337218128 (98.08%)	4170225131 (94.3%)	8620 (0.0%)	2163898395 (48.93%)
CK-3	4716687600	4616704403 (97.88%)	4429135957 (93.9%)	9255 (0.0%)	2310618908 (48.99%)	4580950634	4487856882 (97.97%)	4308143195 (94.04%)	8985 (0.0%)	2239608501 (48.89%)
T1-1	2198706900	2147175838 (97.66%)	2051450078 (93.3%)	6150 (0.0%)	1060872656 (48.25%)	2117567222	2070532352 (97.78%)	1980430347 (93.52%)	5923 (0.0%)	1018163264 (48.08%)
T1-2	2427153900	2367957095 (97.56%)	2258489920 (93.05%)	6754 (0.0%)	1167102364 (48.08%)	2345848123	2291202640 (97.67%)	2187429602 (93.25%)	6534 (0.0%)	1124408977 (47.94%)
T1-3	2465749800	2406086402 (97.58%)	2295907224 (93.11%)	6923 (0.0%)	1192152276 (48.35%)	2381100463	2326232408 (97.7%)	2221927473 (93.32%)	6687 (0.0%)	1147557638 (48.2%)

Table 2 Reads Filter information statistics table

Sample	Raw Reads Num	Clean Reads Num(%)	Read Length	Adapter (%)	Low Quality (%)	Poly A (%)	N (%)
CK-1	33383672	33343154 (99.88%)	150 + 150	2370 (0.01%)	76296 (0.11%)	0 (0%)	0 (0.0%)
CK-2	30345772	30318650 (99.91%)	150 + 150	1888 (0.01%)	50468 (0.08%)	0 (0%)	0 (0.0%)
CK-3	31444584	31410618 (99.89%)	150 + 150	2236 (0.01%)	63460 (0.1%)	0 (0%)	0 (0.0%)
T1-1	14658046	14639024 (99.87%)	150 + 150	708 (0.0%)	36628 (0.12%)	0 (0%)	0 (0.0%)
T1-2	16181026	16160994 (99.88%)	150 + 150	720 (0.0%)	38624 (0.12%)	0 (0%)	0 (0.0%)
T1-3	16438332	16414252 (99.85%)	150 + 150	906 (0.01%)	46348 (0.14%)	0 (0%)	0 (0.0%)

Table 3 Compare ribosome statistics

Sample	Clean Reads Num	Mapped Reads	Unmapped Reads
CK-1	33343154	550600 (1.65%)	32792554 (98.35%)
CK-2	30318650	768910 (2.54%)	29549740 (97.46%)
CK-3	31410618	659792 (2.10%)	30750826 (97.90%)
T1-1	14639024	140038 (0.96%)	14498986 (99.04%)
T1-2	16160994	180378 (1.12%)	15980616 (98.88%)
T1-3	16414252	179954 (1.10%)	16234298 (98.90%)

Table 4 Compare the genome tables

Sample	Total_Reads	Unmapped_Reads	Unique_Mapped_Reads	Multiple_Mapped_reads	Mapping_Ratio
CK-1	32792554	973644(2.97%)	31517599(96.11%)	301311(0.92%)	97.03%
CK-2	29549740	531662(1.80%)	28827721(97.56%)	190357(0.64%)	98.20%
CK-3	30750826	750730(2.44%)	29742072(96.72%)	258024(0.84%)	97.56%
T1-1	14498986	819436(5.65%)	13568195(93.58%)	111355(0.77%)	94.35%
T1-2	15980616	971741(6.08%)	14904541(93.27%)	104334(0.65%)	93.92%
T1-3	16234298	860689(5.30%)	15260205(94.00%)	113404(0.70%)	94.70%

Table 5. All samples tested gene number statistics

Reference Genes	All Known Gene Num
4035	3369 (83.49%)

Table 6. The number of genes detected in each sample was counted

Sample Name	Known Gene Num
CK-1	3249 (80.52%)
CK-2	3265 (80.92%)
CK-3	3268 (80.99%)
T1-1	3239 (80.27%)
T1-2	3244 (80.40%)
T1-3	3210 (79.55%)
